# Analyzing Two-Phase Single-Case Data with Non-overlap and Mean Difference Indices: Illustration, Software Tools, and Alternatives

**DOI:** 10.3389/fpsyg.2016.00032

**Published:** 2016-01-21

**Authors:** Rumen Manolov, José L. Losada, Salvador Chacón-Moscoso, Susana Sanduvete-Chaves

**Affiliations:** ^1^Departamento de Metodología de las Ciencias del Comportamiento, Facultad de Psicología, Universidad de BarcelonaBarcelona, Spain; ^2^Psicología Experimental, Universidad de SevillaSeville, Spain; ^3^Universidad Autónoma de ChileSantiago, Chile

**Keywords:** non-experimental, single-case, data analysis, guidelines, methodological quality

## Abstract

Two-phase single-case designs, including baseline evaluation followed by an intervention, represent the most clinically straightforward option for combining professional practice and research. However, unless they are part of a multiple-baseline schedule, such designs do not allow demonstrating a causal relation between the intervention and the behavior. Although the statistical options reviewed here cannot help overcoming this methodological limitation, we aim to make practitioners and applied researchers aware of the available appropriate options for extracting maximum information from the data. In the current paper, we suggest that the evaluation of behavioral change should include visual and quantitative analyses, complementing the substantive criteria regarding the practical importance of the behavioral change. Specifically, we emphasize the need to use structured criteria for visual analysis, such as the ones summarized in the What Works Clearinghouse *Standards*, especially if such criteria are complemented by visual aids, as illustrated here. For quantitative analysis, we focus on the non-overlap of all pairs and the slope and level change procedure, as they offer straightforward information and have shown reasonable performance. An illustration is provided of the use of these three pieces of information: visual, quantitative, and substantive. To make the use of visual and quantitative analysis feasible, open source software is referred to and demonstrated. In order to provide practitioners and applied researchers with a more complete guide, several analytical alternatives are commented on pointing out the situations (aims, data patterns) for which these are potentially useful.

## Introduction

The evidence-based practices movement aims to provide guidelines for carrying out methodologically sound research in fields such as psychology (Apa Presidential Task Force on Evidence-Based Practice, 2006) and special education (Odom et al., 2005). According to this movement, the studies providing solid evidence need to meet a series of criteria related to how an experimental effect is documented and how generality can be established (Maggin et al., 2014). The first of these aspects refers, among other features of the study, to its design and analysis. In the current work, we focus on two-phase designs that do not meet the criteria established by the What Works Clearinghouse *Standards* (Kratochwill et al., 2010), unless they are part of a within-study replication, as in a multiple-baseline design. Two-phase designs may be weaker, from the perspective of internal validity, but they are still used (e.g., Cordery et al., 2010; O’Neill et al., 2013; Finn and McDonald, 2014; Winkens et al., 2014) and can be useful as pilot studies and also due to the fact that establishing the evidence basis of interventions is related to the replication of results and their integration via systematic reviews and meta-analyses (Jenson et al., 2007). Such reviews can offer a comprehensive summary of findings while trying to avoid publication bias, which would take place when excluding studies on the basis of the design. In that sense, it is potentially useful to report the results of all studies and, afterward, consider whether some studies show no differences or negative results (Kratochwill et al., 2001) or whether there are differences according to the design used or the methodological quality of the study. Actually, Gage and Lewis (2014) suggest that experimental control can be used as a moderator variable in meta-analyses.

In this context, the present paper arises from our conviction that practitioners’ professional practice, mainly aimed to help individual clients, can also contribute to informing fellow professionals about the results of applying certain interventions. In order to make this contribution possible and in order to be able to translate practice into research certain design and analysis considerations are necessary. The current paper mainly aims to answer two specific questions “What can be done to improve the data analysis in my practice so that its results are more useful to the discipline, despite using a sub-optimal design?” and “How can I easily implement some appropriate analytical techniques?” However, design and data analysis should be considered jointly (Brossart et al., 2014) and this is why we first review some aspects related to how the study is conducted.

Regarding the ways in which a study can be considered as providing evidence, a design implemented as a randomized controlled trial is one option, but it is not always feasible. Another alternative is single-case designs, also referred to as N-of-1 trials (Howick et al., 2011). For this latter option, there are several guidelines on how the studies should be carried out (see Smith, 2012; Maggin et al., 2014, for a review). Two of these guidelines are What Works Clearinghouse *Standards* (Kratochwill et al., 2010) and the Risk of Bias in N-of-1 Trials (RoBiNT) scale by Tate et al. (2013). In brief, the optimal features of a single-case study contributing solid evidence are: to use a design allowing for at least three comparisons between conditions (as in multiple baseline, alternating treatments, and ABAB designs; Barlow et al., 2009); to include randomization in the design when assigning measurement times to conditions (Kratochwill and Levin, 2010); to include blinding of the patient, therapist, and assessor; to show high inter-rater reliability when recording the data (especially useful when by means of observation, Cohen, 1960); to apply the intervention as planned (see also Ledford and Gast, 2014, for a discussion on procedural fidelity); the use a repeatable measure for the target behavior; to use an appropriate data analysis procedure; to assess generalization across other behaviors and settings; and to replicate the results.

These requirements reflect the aspects of a study or a professional practice that moderate the extent to which its findings are “solid evidence” and also affect the practitioner’s confidence in the conclusions regarding intervention effectiveness. Accordingly, using a sub-optimal two-phase design such as AB (referred to as “pre-experimental,” Kazdin, 1982, or “quasi-experimental,” Campbell and Stanley, 1966) is a drawback, but it does not necessarily preclude a study from being useful^1^, as there are other characteristics that can increase the credibility in the obtained results. In the present work, we focus on one of these aspects – data analysis – showing how to meet the condition for an appropriate data analysis.

The structure of this article is as follows. First, we comment on the characteristics of non-experimental studies in order to frame a context, where improvements are required (Institute of Education Sciences, 2013). Second, we present an analytical method meeting the criterion for appropriate data analysis; we refer to its strengths, limitations, and alternatives. Third, we apply the analytical method to a real data set. Fourth, we point out several analytically challenging situations and present our own advice to practitioners and applied researchers. With the justification and illustration of the analytical method and the software, we aim to offer practitioners and applied researchers a useful tool, and indications about its alternatives.

## Non-Experimental Studies

Demonstration of causal relations via experimental designs is considered optimal for building the evidence basis of interventions (Kratochwill et al., 2010; Tate et al., 2013), but everyday practice cannot always meet this requirement (e.g., due to time pressure or to the unethical withholding or removal of a potentially beneficial intervention). However, non-experimental studies can still contribute via in-depth assessment of effects, taking into consideration different sources of information (e.g., visual and numerical analyses of the data gathered, the interpretation of the client, his/her significant ones, and the practitioner) and relying on replication.

**FIGURE 1:**
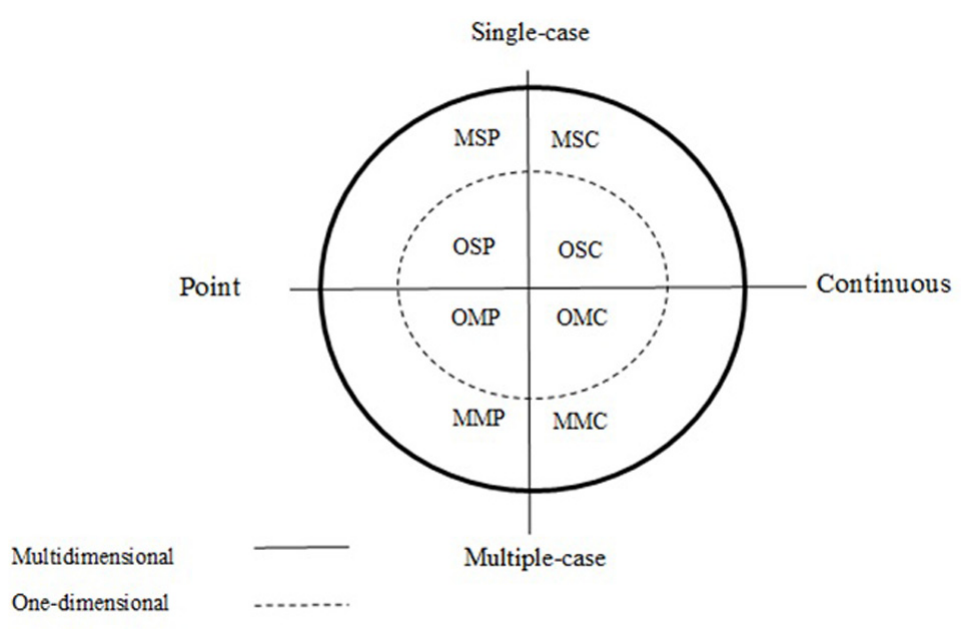
**A classification system for gathering data via observation.** The acronyms of the figure correspond to the initials of the levels of the three components: behavior (multidimensional or one-dimensional), participant (single-case or multiple-case), and time (point or continuous), respectively.

Non-experimental studies consisting only of a pre-intervention and post-intervention condition resemble “natural experiments,” such as disasters or legislation changes, and they also resemble observational studies in which continuous recording of a single individual is taking place (see **Figure 1** representing the taxonomy of observation studies by Anguera et al., 2001, used in Jonsson et al., 2006). Moreover, an experimental multiple-baseline design across behaviors is similar to an observational plan in which several behaviors of the same participant are recorded each time that a video-taped situation is seen by the observers (i.e., a multidimensional observational recording according to Anguera et al., 2001). Another similarity can be seen between a multiple-baseline design across subjects and a multiple-case one-dimensional continuous recording observational plan. However, observational (or non-experimental, in general) and experimental methodology allow reaching different conclusions. Regarding experimental control, the main differences are in: (a) the use of randomization to decide when to introduce and withdraw an intervention, (b) the staggered introduction of the intervention and (c) the replication of effects. Accordingly, in the absence of staggered introduction of the intervention, in an observational study there is less control over alternative explanations of potential behavioral change and the demonstration of intervention effectiveness is not so strong (Kazdin, 1984). Thus, multidimensional single-case continuous observation is not equivalent to multiple-baseline design across behaviors. Moreover, in a natural setting it is usually not possible to choose *at random* when to intervene in order to support internal and conclusion validity (Kratochwill and Levin, 2010). Thus, the conclusions made need to refer to the existence and amount of change in the behavior, but not to the cause for such a change.

## The Analytical Method Explained

The analytical method is grounded on the “data analysis” item of the RoBiNT scale: controversy remains about whether the appropriate method of analysis in single-case reports is visual or statistical. Nonetheless, two points are awarded if systematic visual analysis is used according to steps specified by Kratochwill et al. (2010, 2013), or visual analysis is aided by quasi-statistical techniques, or statistical methods are used where a rationale is provided for their suitability (Tate et al., 2013, p. 629).

Our proposal is to use the option of “visual analysis aided by quasi-statistical techniques,” where the latter are understood as descriptive measures that do not intend to yield statistical significance values due to various reasons. First, visual analysis is not only frequently used, but it is apparently the only kind of single-case data analysis that researchers seem to agree that is necessary (e.g., Parker et al., 2006; Gast and Spriggs, 2010; Kratochwill et al., 2010; Davis et al., 2013; Fisher and Lerman, 2014). Second, the evidence on visual analysis suggests that its exclusive use is potentially problematic (i.e., visual analysis is not sufficient) and techniques increasing the reliability of visual analysis are necessary (Maggin et al., 2013). Third, we consider that certain quasi-statistical techniques with favorable evidence for their performance can be used as natural complements of the commonly used visual analysis, as they share the emphasis on the same main data features (overlap, level, and trend), whereas the visual aids also take data variability into account and allow comparing projected and actual data. Fourth, applied researchers may not be willing to use the more complex statistical techniques whose results are more easily misinterpreted, in case of incomplete understanding of what exactly is being done with the data. Fifth, the use of inferential statistical procedures may not be fully justified in the absence of random sampling (Edgington and Onghena, 2007). Moreover, an inference to a population is not necessarily an aim of idiographic research (Johnston and Pennypacker, 2008) that focuses on the needs and the improvement of the individual clients. Sixth, easy to use software is available for the descriptive statistical procedures recommended here.

## Systematic Visual Analysis

### Rationale

Visual analysis has been and still is popular among professionals in their everyday psychological practice (Robey et al., 1999; Parker and Brossart, 2003) and is still advocated for (Lane and Gast, 2014) and used as a gold standard for assessing quantitative procedures (Wolery et al., 2010). Visual analysis has been considered both appropriate and sufficient for data gathered longitudinally (Michael, 1974). However, this sufficiency has been defended only for experimental studies (Sidman, 1960), which points at the need for complementing it with a quantitative procedure.

Tate et al. (2013) advise for systematic visual analysis and it necessarily starts with assessing the baseline, specifically, whether the intervention can be introduced or it should be postponed until stability is reached (Barlow et al., 2009). Alternatively, deterioration in the behavior of interest would suggest even more clearly the need for intervention. In that sense, deterioration is not expected to interfere with subsequent conclusions about intervention effectiveness (Kazdin, 1978), given that it allows exploring whether an intervention reverts the situation. Nonetheless, it is possible to assess intervention effectiveness even when the behavior is already improving before the intervention itself, as it will be shown later.

The specific data aspects, which are foci of attention, are the amount of overlap between data in the different conditions, within- and between-phase variability, slope and level change (SLC; Kratochwill et al., 2010; Lane and Gast, 2014). A more objective assessment of the degree to which data share the same values (i.e., overlap), whether levels and trends are similar across conditions, and whether data become more stable or more variable after the intervention can be done using visual aids instead of relying on naked-eye impressions. Finally, visual analysis focuses on the whole data pattern (Parker et al., 2006) in order to assess whether it resembles the expected one, that is, a consistent improvement only during intervention. Kratochwill et al. (2010) summarize the overall assessment as a comparison between projected and actually obtained measurements. Specifically, in two-phase designs, it is relevant to project the baseline (in case it is stable or presents trend stability) into the intervention phase and compare this projection with the real treatment phase data.

### Potentially Useful Tools

**FIGURE 2:**
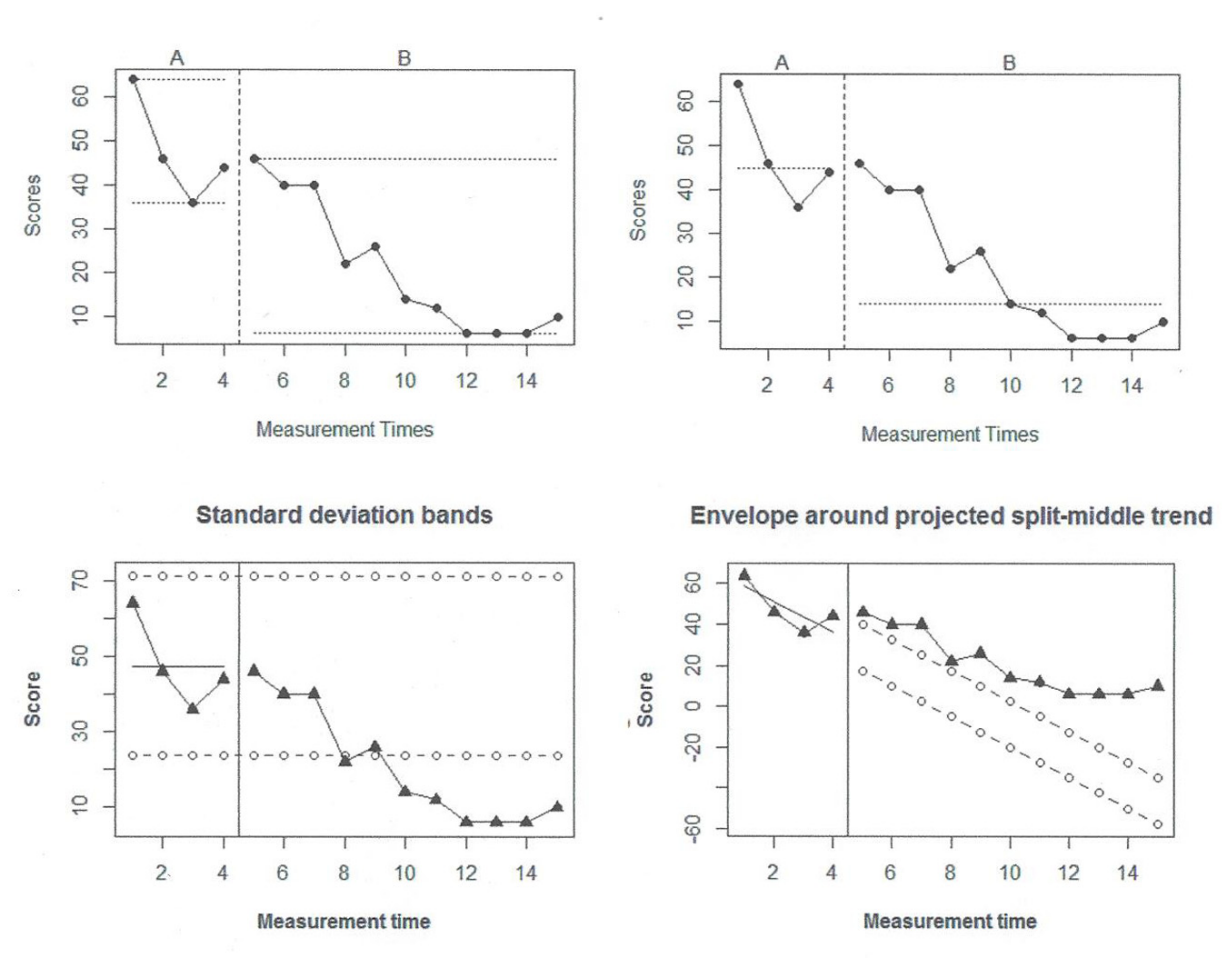
**An illustration of visual sides using Taylor and Weems (2011) data on a participant called Elizabeth.** Upper left panel-range bars. Upper right panel-medians. Lower left panel-2-standard deviation bands. Lower right panel-stability envelope around split middle trend.

The assessment of overlap can be done using visual aids, such as range lines, as provided by the SCDA plug-in (Bulté and Onghena, 2012^2^) for R-Commander. The upper left panel of **Figure 2** shows an example with the data reported by Taylor and Weems (2011) for a participant called Elizabeth. This graph suggests a minor overlap between the observations. Regarding the assessment of changes in level, the same software can be used to superimpose, for instance, the median of the behavioral observations in the pre-intervention and post-intervention conditions.The upper right panel of **Figure 2** shows an example with the same data and suggests that there has been a reduction in the level of target behavior. However, the median is not very useful for the post-intervention observations in which there is a clear downward trend.

Regarding the assessment of changes in slope, two situations should be considered: when pre-intervention data are stable and when baseline data show an upward or downward trend. In case of stability, it is possible to use the stability envelope (Lane and Gast, 2014) or the two-standard deviations band used in statistical process control (Callahan and Barisa, 2005). The two-standard deviations band implies computing the average of the data for a specific condition and representing it with a solid line. The standard deviation of the same data is also computed and two dashed lines are represented: one located two standard deviations below the mean and the other two standard deviations above. The basis of this procedure is that, for a normally distributed variable, few points (less than 5%) are expected to be out of these limits in case there is no change in the behavior with the introduction of the intervention. However, we suggest using it only as visual aid and not as a formal statistical procedure, as the data cannot be reasonably assumed to be normal, continuous, or independent. This visual aid is implemented in R Core Team (2013) code^3^ that only requires inputting the data and specifying the number of pre-intervention observations. As an example see the lower left panel of **Figure 2**, indicating that the reduction in behavior is beyond what is expected only by random variability as there are multiple observations with values smaller than the lower limit.

In case the pre-intervention data show a trend, it is necessary to compare the projection of this trend and the actually obtained measurements (Kratochwill et al., 2010). For that purpose, there is another potentially useful R code^4^ which allows applying the stability envelope to the trend line: (a) estimating split-middle trend (Miller, 1985), (b) projecting it into the next phase, and (c) constructing an envelope around it. The envelope can be constructed on the basis of the baseline median^5^, so that the lower limit is located 25% of the median below the estimated split-middle trend and the upper limit at the same distance above it (Lane and Gast, 2014). In case 80% of the data are within those limits, this would indicate trend stability, that is, it would suggest that no change in slope has been produced with the introduction of the intervention. For using this code only data input is required before copy-pasting it in R. The lower right panel of **Figure 2** shows an example with Elizabeth’s data. Given that the projected trend and its stability envelope are lower than the actual observations, this is the only piece of graphical information that does not suggest improvement in the behavior, but practitioners should be cautious when trend is estimated from as few as four observations and when it is projected farther away in time into values that are out of the range of possible measurements (Parker et al., 2011b).

Another aspect assessed is whether the introduction of the intervention has led to an immediate change in the behavior. Moreover, the duration of the change (maintained or transitory) is also taken into account in order to evaluate the strength of the intervention. A structured guide on visual analysis is offered by the What Works Clearinghouse *Standards* (Kratochwill et al., 2010; see also the application and a scoring procedure by Maggin et al., 2013) and by Lane and Gast (2014).

### Limitations

Despite these guidelines on visual analysis, there are still no soundly based formal decision rules for all data aspects that are visually assessed (Kazdin, 1982) and objective and replicable outcomes are also missing (Robey et al., 1999). These two drawbacks might be among the reasons for the frequently reported inadequate performance of visual analysts (Gibson and Ottenbacher, 1988; Ottenbacher, 1990; Danov and Symons, 2008; Ximenes et al., 2009; see also Ninci et al., 2015, for a recent meta-analysis reporting insuficient interrater agreement, especially among single-case experts). Moreover, the visual analysts’ decisions are not directly useful for documentation or for meta-analysis (Busse et al., 1995), which would allow establishing the evidence basis for interventions (Jenson et al., 2007), especially as generalization in single-case studies depends on replication^6^ rather than on random sampling and statistical inference. As a result of these limitations, there is a consensus that visual and quantitative analyses should be used jointly (Franklin et al., 1996; Fisch, 2001; Houle, 2009; Harrington and Velicer, 2015).

## Quantitative Analyses Recommended

Our choice of procedures [non-overlap of all pairs (NAPs); Parker and Vannest, 2009 and SLC; Solanas et al., 2010a] is based on the six criteria detailed below, although alternative quantifications are provided later in this article.

### Criterion 1: Simple to Compute

The techniques are relatively simple to compute and offer straightforward interpretations for practitioners who are not experts in statistics (as the Institute of Education Sciences, 2013, suggests). The calculation does not entail statistical decisions about the likelihood of obtaining such a large difference under the null hypothesis. This criterion also relates to the need for easily trainable procedures (Fisher et al., 2003).

### Criterion 2: Complementary to Visual Analysis

This criterion is related to the popularity of visual analysis among practitioners (Parker and Brossart, 2003), which makes necessary to develop and promote suitable complements to it. NAP and SLC are actually based on relevant visual criteria (i.e., data overlap, change in slope and in level) and thus potentially useful as complements^7^. Specifically, visual inspection can be used to assess the adequacy of the baseline as a reference for comparison. The change identified visually can then be quantified in an objective manner. The numerical values also offer information that can be communicated among researchers and professionals and used for further analyses with different analytical techniques or as part of research synthesis (e.g., NAP was used in the meta-analysis by Jamieson et al., 2014, whereas the new developments on SLC make possible its comparability across studies; Manolov and Rochat, 2015).

### Criterion 3: Synergic Application

Wolery et al. (2010) criticized non-overlap methods for omitting relevant data aspects such as level, trend, and stability or variability: SLC partially addresses this issue and it also responds to Beretvas and Chung’s (2008) suggestion for quantifying separately level and slope change. Moreover, SLC yields unstandardized results, which help assessing the practical importance of the behavior change when using meaningful measures (Grissom and Kim, 2012) such as the number of tantrums or the number of self-injurious behaviors. In contrast, NAP is bounded, which allows comparisons and quantitative integrations. Thus, NAP and SLC can be used jointly as they provide different information. Specifically, NAP is an ordinal measure (Solomon et al., 2015) that does not distinguish between conditions once complete overlap is achieved. In contrast, SLC can be used even in absence of overlap to quantify how different the measurements belonging to different phases are.

### Criterion 4: Absence of Assumptions and Restrictions of Use

The procedures used here do not make explicit *a priori* assumptions about independence or homoscedasticity of the data, as serial dependence is likely to present in data obtained from the same individual (Matyas and Greenwood, 1996). There are also no specific design requirements.

### Criterion 5: Appropriate Performance

In relation to the previous point, there is evidence that their performance is appropriate for a variety of single-case data patterns (Manolov et al., 2011). NAP is a suitable indicator when data is stable and even when data is variable. In contrast, in such situations visual analysis is more difficult to perform and means and medians are not informative and trends are not estimated with precision. On the other hand, NAP is not suitable when the data show improving trend, but SLC can be applied in such a situation – this complementarity relates to Criterion 3 “Synergic application.” SLC is useful for separately quantifying the change in level and the change in slope in potentially meaningful terms. In relation to this criterion, it is important to discourage the use of methods for comparing conditions that have been shown not to perform appropriately, such as the binomial test applied after the split-middle method (Crosbie, 1987) which does not control for Type I error rates, ITSACORR which presents modeling flaws (Huitema et al., 2007), or the C-statistic (Young, 1941; Tryon, 1982; used by Fabio et al., 2013), which is actually an estimator of autocorrelation (DeCarlo and Tryon, 1993).

### Criterion 6: Reduced Likelihood of Misinterpretation

Using descriptive measures like the ones provided by NAP and SLC makes it less likely for applied researchers to make inferences, which would be statistically incorrect in absence of random sampling of the participant or of the behavior of interest (Barlow et al., 2009). We consider that inferential statistical techniques are more susceptible to being misunderstood and to prompt researchers to make dichotomous decisions (Cohen, 1994) about intervention effectiveness or behavioral change. In case inference is desired, we recommend causal inference, instead of population inference, in line with the recommendations by Heyvaert et al. (2015).

### Non-overlap of All Pair

Non-overlap of all pairs is an improvement of the Percent of non-overlapping data commonly used for quantifying the degree to which the measurements pertaining to each phase share the same values (Scruggs and Mastropieri, 2013). It represents the number of non-overlapping data relative to all possible comparisons and it is actually identical to the non-parametric version of the probability of superiority (Grissom, 1994), which is related to the common language effect size (McGraw and Wong, 1992). When a decrease in the behavior is expected, as in the example provided later, the formula for this indicator can be written as (#(*X*_*pre*(*i*)_ > *X*_*post*(*j*)_) + 0.5#(*X*_*pre*(*i*)_ = *X*_*post*(*j*)_))/*n_pre_n_post_* where *X_pre_* and *X_post_*, which represent the values of the pre-intervention and post-intervention phases, respectively, with *i* = 1,2,⋅⋅⋅,*n_pre_* and *j* = 1,2,⋅⋅⋅,*n_post_*, and # denotes the number of times that the inequality or the equality is true. Given that each data point of the pre-intervention phase is compared to a data point from the post-intervention phase there is a total of *n_pre_n_post_* comparisons, where *n_pre_* and *n_post_* denote the number of measurements in the first and second phase, respectively. In each of these comparisons, a non-overlap occurs when a post-intervention measurement represents an improvement over a pre-intervention measurement, with ties counting as half a non-overlap. To obtain the index value, the number of non-overlapping pairs is divided by number of comparisons. This value can be interpreted in two different ways. One the one hand, it represents the proportion of comparisons for which intervention phase data improve baseline data. On the other hand, it can be conceptualized as the probability that a randomly selected post-intervention data point will improve (here, be smaller than) a randomly selected pre-intervention data point. The NAP can be computed via the online calculator http://www.singlecaseresearch.org/calculators/nap by Vannest et al. (2011), where it is only necessary to enter the data from the different conditions in separate columns. It is also part of the output (“A vs. B” comparison) of the R code for Tau-U https://dl.dropboxusercontent.com/u/2842869/Tau_U.R (Brossart et al., 2014), which requires loading a data file with a single comma-separated column including “Time” (1, 2, …, *n_pre_+n_post_*), “Score” (denoting the measurements) and “Phase” denoting the condition (*n_pre_* times the value of 0 followed by *n_post_* times the value of 1).

### Slope and Level Change

Slope and level change quantifies two aspects of behavior’s evolution after a change in the conditions: change in slope and change in level. Actually, this procedure first estimates pre-intervention linear trend (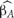) as the average of the differenced first phase measurements, that is, 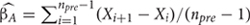. Baseline trend is thus the average increase (or, if negative, decrease) from one baseline measurement occasion to the next one. This estimation can inform about the characteristics of the data before an intervention is introduced. Moreover, baseline trend is removed from the whole data series so that it does not affect the quantification of the effects of the intervention. Technically, each data point is corrected according to its position in the series of observational sessions. This initial step allows for applying an intervention even when the theoretically undesirable linear improvement is present already during the assessment period. Thus, SLC would show whether there is an effect of the intervention beyond the initial improvement. After the correction it is assumed that the pre-intervention phase shows zero trend (i.e., stable data) and thus the trend present in the post-intervention phase actually represents an effect (i.e., a change in slope). This effect is estimated in the same manner as in the initial step, that is, as the average of the differenced (and already detrended) post-intervention measurements: 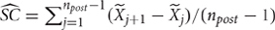, where 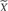 represent detrended values (i.e., after eliminating pre-intervention trend), instead of the original measurements. Therefore, the intervention phase estimate of trend presents the average increase (or, if negative, decrease) from one intervention phase measurement occasion to the next one, after controlling for baseline linear trend. For instance, the slope change estimate reflects the average decrease in the number of tantrums in a child with each successive post-intervention measurement, that is, a progressive change.

Once slope change is estimated, post-intervention trend is removed in order to obtain a net estimate of the change in level. This way of proceeding is similar to what is done in ARIMA models, before obtaining a quantification of change in level (see Harrington and Velicer, 2015). Net change in level is estimated as the difference between the average of the corrected post-intervention measurements and the average of the corrected pre-intervention measurements. The expression for this step is 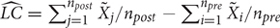, where 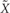 represents post-intervention measurements with both pre-intervention trend and post-intervention trend (i.e., slope change) removed and 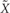 represents pre-intervention measurements with pre-intervention trend removed. The net level change estimate quantifies, for instance, the average decrease of tantrums in a child after the intervention, once slope change has been taken into account. Thus, it can be conceptualized as a quantification of an abrupt and maintained effect. The SLC can be computed using R code https://dl.dropboxusercontent.com/s/ltlyowy2ds5h3oi/SLC.Ror via the R-Commander Plug-in offering point-and-click menus, available at http://cran.r-project.org/web/packages/RcmdrPlugin.SLC/index.html. For obtaining the numerical results and a graphical representation of the original and detrended data, both options only require inputting the values of the observations and specifying the pre-intervention phase length.

## Alternatives for Quantitative Analysis

There is currently no consensus on which the optimal quantitative procedure for single-case designs is (Kratochwill et al., 2010; Smith, 2012), as the RoBiNT scale also reflects (Tate et al., 2013). For a comprehensive review of most currently available techniques the interested reader should consult the state-of-the-art information provided in the Special Issues of the *Journal of School Psychology* in 2014, volume 52, issue 2 (e.g., Shadish et al., 2014; Swaminathan et al., 2014) and of *Neuropsychological Rehabilitation* also in 2014, volume 24, issues 3-4 (e.g., Borckardt and Nash, 2014; Brossart et al., 2014; Heyvaert and Onghena, 2014). Here, we provide brief comments on the strengths and limitations of several analytical alternatives, which in some cases may be more appropriate than NAP and SLC included in the analytical method suggested.

Considering specifically observational studies in which data is recorded continuously within a session, it is possible to follow an analytical approach different from the one used in single-case designs, namely, to apply sequential analysis to explore whether the occurrence of some behaviors make more or less probable that other behaviors take place (Bakeman and Quera, 2011). Additionally, longer series of data gathered across time can be analyzed using Markov chains or analyses of rhythm, according to the aims of the study (Suen and Ary, 1989).

Starting our discussion from procedures similar to the ones included in the analytical method, Tau-U (Parker et al., 2011b) is closely related to NAP and it is preferable when pre-intervention trend is present in the data. For both Tau-U and NAP *p*-values have been offered, although their basis has not clearly been explained in the presence of autocorrelation. However, Tau-U is interpretatively and computationally less straightforward than NAP (i.e., Criterion 2 “Complementary to visual analysis” is met to a lesser extent). For instance, even in case a baseline trend is generally deteriorating, if there is a single improving value in the baseline phase, as compared to a previous baseline data point, this would reduce the value of the non-overlap index. Thus, in case trend is not reasonably clear, Tau-U can be an excessively conservative procedure (i.e., it would overcorrect). Furthermore, more evidence is required on its performance (thus the abovementioned Criterion 5 “Appropriate performance” is not fully met, as Parker et al., 2011a,b, offer only applications to real data, but no simulation study).

Regarding procedures quantifying average differences, similar to the SLC, the *d*-statistic (Shadish et al., 2014) has to be mentioned. We highlight here the *d*-statistic developed by Shadish et al. (2014), which has been created specifically for single-case designs rather than the *d*-statistic described by Busk and Serlin (1992; approach one^8^), recommended by Beeson and Robey (2006), for two reasons: (a) the latter is an adaptation of the group designs indicator and does not take into account autocorrelation, while it has been shown to be somewhat affected by autocorrelation (Manolov and Solanas, 2008); and (b) its sampling distribution in single-case studies is unknown (Beretvas and Chung, 2008). In contrast, the *d*-statistic developed by Shadish et al. (2014), offers a standardized measure of the mean difference with a solid statistical basis offering the possibility to estimate the index variance for future meta-analyses. So far, it has been developed for AB, reversal (e.g., ABAB) and multiple-baseline designs and assuming that pre-intervention data is stable, assuming that within-case residuals and between-case variation do not change over time. Thus, this procedure fails in terms of Criterion 4 “Absence of assumptions and restrictions of use.” Some potential drawbacks include: (a) its computation requires several cases per study; and (b) the calculations are potentially difficult to understand by applied researchers with less statistical knowledge and require the use of software, such as the R code provided in the appendix of the Shadish et al. (2014) paper. Hence, the *d*-statistic is preferable to SLC when there is more than one participant per study and the aim is to obtain a standardized measure, but it is not suitable when pre-intervention trend is present and when the focus on a specific client.

Generalized least squares regression analysis (Swaminathan et al., 2014) also enables computing an effect size index. Its strengths include the fact that it can take into account changes in level and in slope (although they are quantified as part of the same overall indicator, unlike SLC), the versatility in modeling (e.g., controlling for linear and non-linear trends), and that it deals explicitly with autocorrelation. However, autocorrelation estimation has been shown to be problematic (Solanas et al., 2010b) and the analytical procedure requires several steps, some of them taking place iteratively (i.e., Criterion 1 “Simple to compute” is not met). This procedure is applicable to longer data series for which autocorrelation can be estimated with greater precision. Moreover, we recommend that practitioners work together with a statistician, so that the analysis can be properly run. Brossart et al. (2006) compared the agreement between visual analysis and several regression-based approaches and the best performer in this terms (related to Criterion 2 “Complementary to visual analysis”) was Allison and Gorman’s (1993) method, which is however affected by autocorrelation (Manolov and Solanas, 2008). The generalized least squares approach was not yet proposed by the time Brossart et al. (2006) conducted their study and more evidence is necessary to assess its performance.

Multilevel models are an extension of piecewise regression and can be used to model several data aspects (e.g., trend, autocorrelation, heterogeneous data variability across phases) and they yield estimates of the change in the same measurement units as the target behavior and their statistical significance (Moeyaert et al., 2014a). The main drawbacks of multilevel models are the problematic estimation of variance (Ferron et al., 2009), their relative complexity for applied researchers with less statistical knowledge and the fact that they the replication of the intervention in several participants. Actually, such a complex procedure is more suitable for more complex design structures that the two-phase AB (Moeyaert et al., 2014b). Finally, most implementations of this analytical procedure have been done in commercial software (e.g., Moeyaert et al., 2014a include SAS code in their article).

An effect size index can also be computed from interrupted time series analysis via ARIMA (autoregressive integrate moving average) models, which allow controlling for trend and autocorrelation (Simonton, 1977). The main difficulties of this option are the need for long data series and the problematic initial model identification step. However, there have been suggestions for using some general models that make model identification unnecessary (Harrop and Velicer, 1985). A recent application of ARIMA models has shown that these can be applied to two-phase data, but there might be convergence problems and, more importantly, the agreement with visual analysis is low (Harrington and Velicer, 2015). We consider that this latter drawback and the relative complexity of the technique make it less attractive to applied researchers with no statistical expertise.

Statistical significance (i.e., *p*-values) can be estimated for *d* and the generalized least squares procedure on the basis of the comparison between the test statistic and a theoretical reference (the sampling distribution) and allows making inference about the population from which the individual was drawn. In contrast, randomization tests (Heyvaert and Onghena, 2014) yield a *p*-value on the basis of a comparison between the test statistic and an empirical reference –the randomization distribution. In the current context of two-phase studies, this reference is the distribution of the test statistic values quantifying the difference between the two conditions for each possible intervention start point (i.e., for each possible way in which the data series can be split into two; Edgington, 1980). For this analytical option the inference is restricted to the case studied, referring to the likelihood of obtaining such a large difference in case the intervention was ineffective. Randomization tests are versatile in terms of test statistic to use (e.g., it can be an effect size such as a non-overlap index) and offer flexible options for dealing with different situations (e.g., Levin et al., 2012). However, the necessary randomization as part of the data collection process is both a strength (Kratochwill and Levin, 2010) and a limiting characteristic (Fisher and Lerman, 2014) in a clinical setting (i.e., Criterion 4 “Absence of assumptions and restrictions of use” is not met). Moreover, in certain conditions Type I error rates are not controlled (Manolov et al., 2010). Randomization tests can be recommended when the aim is to obtain statistical significance and the point(s) of change in the conditions can be chosen at random. Randomization tests are also accompanied by freely available software (Bulté and Onghena, 2013; Levin et al., 2014).

Another procedure using an empirical reference distribution is simulation modeling analysis (SMA; Borckardt and Nash, 2014). In SMA, data are generated with the same autocorrelation as estimated from the data, but with no difference between the conditions, thus representing the null hypothesis of identical behavioral level across conditions. The *p*-value represents the likelihood of the outcome, computed as a point biserial correlation between the measurements and a dummy variable representing the condition (0 = without intervention, 1 = with intervention). This approach is intuitive, takes autocorrelation into account, and it can be implemented via the software available freely at http://clinicalresearcher.org/software.htm. However, so far the evidence on its performance (i.e., Criterion 5 “Appropriate performance”) is not sufficient. Finally, as the focus of is put on the *p*-value, which may enter in conflict with Criterion 6 “Reduced likelihood of misinterpretation.”

Whereas SMA uses Monte Carlo methods or bootstrap for generating samples and estimating the likelihood of the value of test statistic in case there is not difference between conditions, bootstrap has also been suggested for single-case as a way of reducing bias and estimating standard errors (McKnight et al., 2000) and specifically for estimating confidence intervals of regression-based *R*-squared values (Parker, 2006). This option has not received much attention lately and it is unclear whether applied researchers would be willing to use it.

Another computer-intensive option could be the Monte Carlo based method for modeling non-linearity proposed by Theiler et al. (1992). However, modeling non-linear patterns can also be achieved without prior knowledge and without the need to specify a model, by using local regression (LOESS; Jacoby, 2000; Solmi et al., 2014). We consider LOESS to be more practical for applied researchers than the Theiler et al. proposal. Moreover, randomization tests are also more parsimonious as they require no assumptions about the process generating the data or about random sampling. Actually, Theiler et al. (1992) mention this option as rank statistic approach for obtaining *p*-values. Randomization test offer the advantage of not only mimicking the preserved data features (such as mean and standard deviation), as expressed by Theiler et al. (1992), but they actually preserve the whole data series and its order, taking advantage of the different possible moments of change in phase, when such moments are determined at random.

**FIGURE 3:**
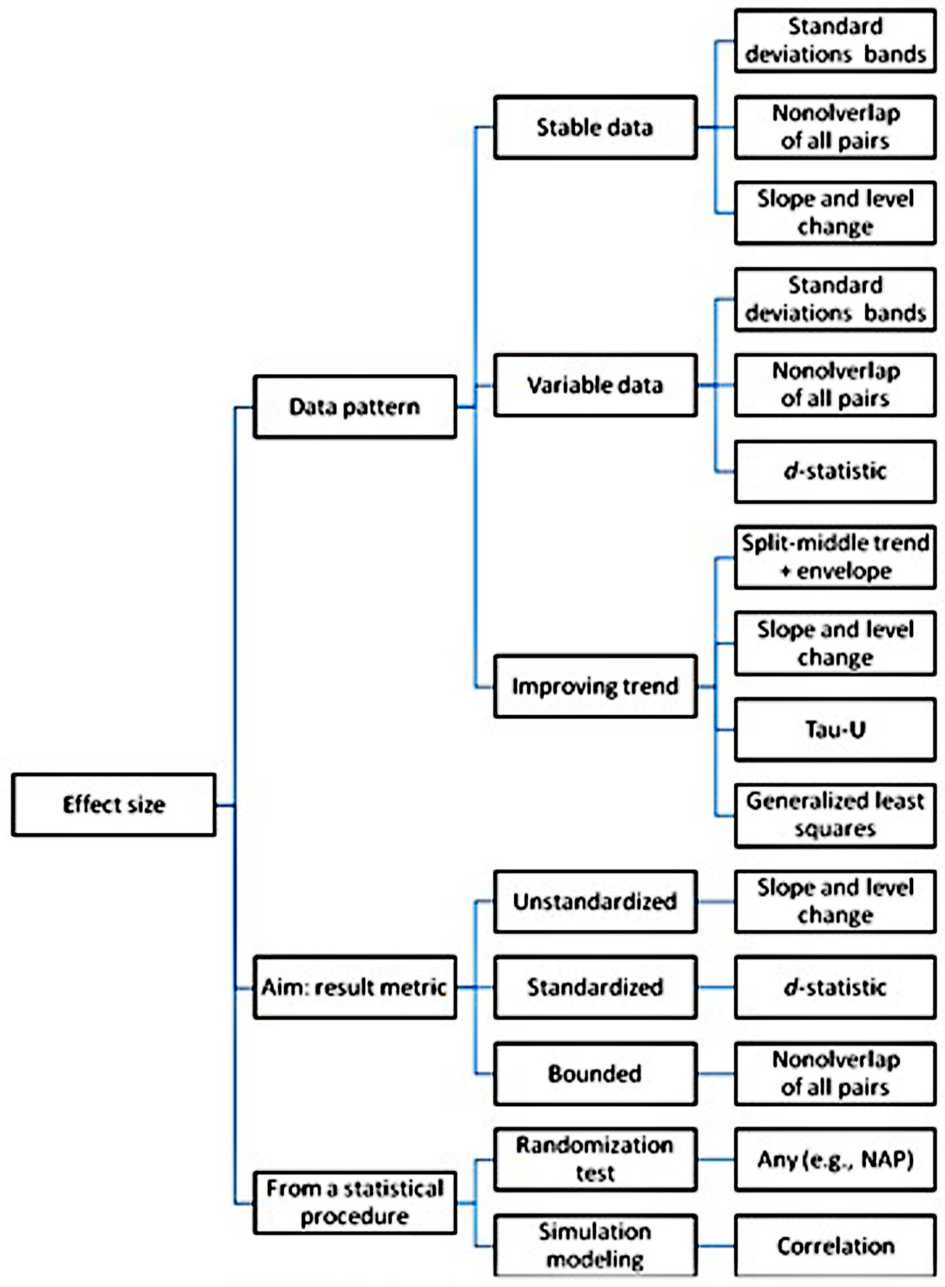
**Graphical (simplified) summary of the recommendations regarding the use of several analytical techniques for single-case experimental and pre-experimental designs**.

A simplified summary of these general recommendations regarding the use of the analytical techniques can be found in **Figure 3.**

## Intervention Effectiveness is Not Only Data Analysis

Assessing the relevance of an intervention cannot be constrained solely to visual and descriptive or inferential statistical analyses. It is important to assess aspects such as quality of life (Kendall, 1999), whether the behavior has moved from dysfunctional to functional ranges (Kazdin, 1999), without forgetting subjective evaluation (Hugdahl and Ost, 1981). Regarding the latter, Kratochwill and Levin (2010) highlight the need to get to know the perceptions of the client and of significant others. According to the specific context being studied, these significant others would be the family members (parents, siblings, marital partner), the teacher, the coach, or the boss (as figure with a higher hierarchical role), and friends, classmates, or colleagues (at the level of “peers”). Kazdin (1984) has referred to these groups of people as “paraprofessionals,” as they help detecting the behavior that requires intervention and they can also be the agents reinforcing the behavior of interest (e.g., a mother reinforcing a child’s disruptive behavior by paying attention to it) or producing stimuli for discriminating conditions in which certain types of behavior are desirable (e.g., a boss may encourage jokes with one type of clients and more distant behavior with others).

## The Analytical Method Applied

**FIGURE 4:**
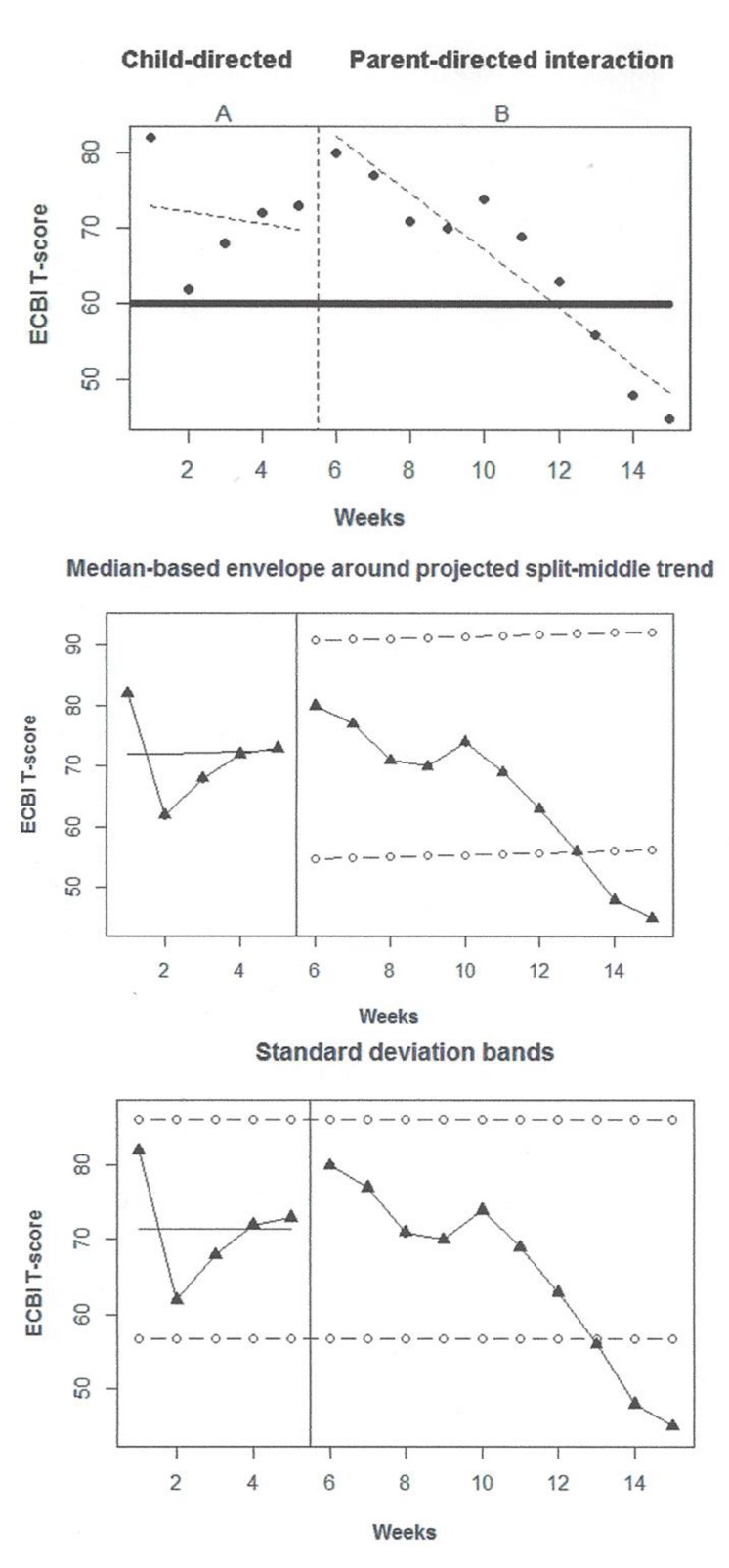
**Graphical representations of the Bagner et al. (2009) data gathered through observation in the family context: upper panel – trend lines; middle panel – split middle and trend envelope; lower panel – standard deviation bands**.

In the present section, we will illustrate the application of the analytical method and the information that can be obtained via visual and quantitative analyses, while also considering substantive criteria. This application focuses on the family context, where it is common to gather data before and after an intervention (Crane, 1985). One of the empirically supported interventions in this context is the Parent Child Interaction Therapy (PCIT; Eyberg et al., 2008), which has been reported to increase positive parent behavior and reduce child behavior problems (Borrego et al., 2006). For the current example, the data gathered by Bagner et al. (2009) will be used. The participants are a 23-months-old premature-born child displaying difficult behaviors and his mother. The application of the PCIT focuses on teaching parenting skills in order to improve the interaction with the child and to decrease his externalizing behavior. Teaching takes place in two phases. First, child-directed intervention (CDI) takes place. It is similar to play therapy: the child is the leader and the parent has to learn how to act positively (e.g., praising the child, imitating the child’s play). Second, parent-directed intervention (PDI) phase occurs. It is similar to clinical behavior therapy: the parent is more directive and has to improve her way of disciplining so that a greater compliance is achieved. In order to assess intervention effectiveness, several sources of information are used: parent reports provided via inventories, observation of the parent–child interaction, and physiological measurements. In the running example, we focus on the parent weekly reports obtained via the Intensity scale of the Eyberg Child Behavior Inventory (ECBI; Eyberg and Pincus, 1999) on disruptive behavior, although a complete assessment entails exploring whether all available information converges to the same conclusion. The Bagner et al. (2009) ECBI data were chosen here given that there is a cut-off point at a T-score of 60 which indicates clinically significant results and eases the interpretation in substantive terms. The data gathered^9^ on the ECBI scale are represented on **Figure 4**. The upper panel contains ordinary least squares trend lines provided by the SCDA plug-in for R, the middle panel contains split-middle trend for the first phase, and the lower panel represents the application of the two-standard deviations band fit to the first condition’s data and projected into the second one.

Firstly, when visually inspecting the data, it has to be kept in mind that both phases are treatment phases and thus in both some reduction in child’s behavior is expected and desired. Moreover, it has to be taken into account that the pre-treatment (i.e., actual baseline) value is 82, equal to the first CDI phase measurement. At the beginning of the first phase there is actually a reduction, but then a new increment starts. Considering this alternating pattern the CDI does not seem especially effective. Given the amount of variability in the first phase, neither the central tendency measure (mean represented on the lower panel of **Figure 4**), nor the different types of trend fitted (upper and middle panel) seem to represent the data well-enough. This can hamper the comparison between this condition and the subsequent one.

Once the intervention is introduced, there is apparently a decrease in the ECBI score on disruptive behavior. The downward trend is stable, as shown by the good fit of the ordinary least squares regression line to the data (upper panel of **Figure 4**). For such data it is not meaningful to discuss level or variability around a mean or a median level; actually variability is only assessed looking at the (small) distance of the measurements from the fitted trend line.

Comparing the two phases in terms of overlap, the values in the beginning of the PDI-phase are similar to the ones in the CDI-phase, but not so in the end. Comparing levels is not meaningful. Comparing trends is hindered by the lack of fit of the trend lines to the CDI data, but if we focus on the last four (out of five) CDI measurements, there is a deterioration that is reverted with the introduction of the PDI: thus a change in slope has taken place. The comparison between projected and actual data is done in two ways, projecting the baseline mean with limits based on the baseline standard deviation and projecting the split-middle trend line with limits based on 25% of the baseline median. In this case, both approaches lead to a very similar graphical representation, which is well-aligned with the conclusion that the last PDI data points are clearly lower that what would be expected (i.e., values within the limits) in case there was no difference between the two interventions. Additionally, we should consider that Bagner et al. (2009) collected a post-treatment measurement equal to 38 – a value even lower than the last PDI-phase measurement and so the downward trend seems to continue, which could be interpreted as maintenance of the effect.

Secondly, regarding quantitative analyses, the NAP performs 50 comparisons, given that *n_pre_* = 5 and *n_post_* = 10, in which there are 19 full overlaps, that is, 19 cases in which a CDI datum is better (here, lower) than a PDI measurement, 0 ties, and 31 cases in which a PDI measurement is better than a CDI data point. (Lower rather than greater values are considered as overlaps, given that the aim is to reduce the disruptive behavior and thus also the ECBI T-score.) The value yielded by NAP is 62.00%, which can be interpreted as the percentage of PDI measurements that improve the CDI measurements. Therefore, the index does not suggest that the change is especially salient, given that the value is only slightly higher than the one expected by chance (50%) and it is within the range of values (0–65%) denoting small effect according to Parker and Vannest (2009). However, it has to be considered that this may be due to the fact that the effect is delayed. The data pattern is not specifically easily analyzed by the SLC either. The procedure estimates the CDI-phase trend as -2.25, which represents an average of approximately two T-score units reduction for each CDI measurement time. However, this value does not reflect the visual impression, provided that this phase shows a specific kind of variability (i.e., an alternating pattern). Correcting for this initial phase trend, the slope change estimate is -1.64, that is, nearly two T-score points average reduction for each PDI measurement time. This quantification reflects to some extent the visual impression of slope change. SLC’s estimate of the net change in level is positive, 18.15, which contrasts with the visual impression of the graphed data.

Thirdly, focusing on substantive criteria, Bagner et al. (2009) summarize their results in terms of improved parent practice and increased child compliance. In fact, while the former result stems from observation and evaluation by the authors, the latter is based in reports from the parents (i.e., the paraprofessionals). Regarding the ECBI scores, the last three scores during the PDI phase fall out of the clinical range, indicating that a practically significant change in behavior of the child has taken place. Interestingly, these same three scores also fall out of the two-standard deviations band and out of the split-middle trend stability envelope represented in the middle and lower panels of **Figure 4**. To complement this assessment, the authors report that at a 4-months follow-up the results of the ECBI remained in the normal range (the value was 47), which increases the confidence in the importance of the behavioral change. Finally, it should be noted that Bagner et al. (2009) comment explicitly the “inability to conduct statistical analyses” (p. 475), which suggests that informing applied researchers about analytical options for two-phase single-case designs, as we intend with the current paper, is a timely endeavor.

The main conclusion of this application of the analytical method is that visual analysis is necessary for focusing at different aspects of the data, such as an unstable baseline which is not well-represent by mean or trend lines, a somewhat delayed slope change, and a considerable amount of overlap only in the beginning of the second condition but not at the end. The variability and relative shortness of the first phase (although it meets the current standards of five measurements; Kratochwill et al., 2010) have to be kept in mind when comparing it to the measurements obtained in the subsequent condition. In the current case, the visual aids reflected this variability and suggested a similar conclusion as the one based on substantive criterion expressed as a cut-off point. All this information is critical for interpreting correctly the numerical yielded by descriptive statistical procedures. Actually, we preferred to use a data set that is challenging for the quantitative analyses in order to alert applied researchers on the need to interpret numerical values with caution and to use all information available; we also wanted to avoid doubts about the data being picked up only to show the quantification in a positive way (Fisher and Lerman, 2014). Finally, the follow-up measures, the parent-report and the physiological measures recorded by Bagner et al. (2009) also contribute to building solid conclusions. The two-phase design may not be sufficient for establishing a causal effect in a scientifically sound way, but there is enough information pointing at the clinically important reduction of problematic behavior.

## Discussion

The present work focused on the question of what can be done to improve the data analysis in studies/practices using sub-optimal designs in such a way that results are more useful to the discipline. We recommended an analytical method consisting of structured visual analysis complemented with descriptive statistical procedures, while also keeping in mind substantive criteria (i.e., the opinion of the individuals involved in the process: family members, teachers, peers, coworkers, or supervisors). On the one hand, quantifications are useful for summarizing different aspects of the data and making the results available for subsequent meta-analysis. On the other hand, visual analysis is required for gaining an in-depth knowledge of the data and for assessing the adequacy of any specific quantitative procedures, due to the lack of consensus regarding the most appropriate technique (Tate et al., 2013).

A second question concerned the availability of tools for implementing the procedures proposed as part of the analytical method. We have mentioned, referenced, and illustrated the output of several tools implemented in the freeware R. Some of them are based on clickable menus, whereas others only require inputting the data before copying and pasting the code. The availability of software is crucial for eliminating the errors in obtaining the numerical and graphical results and in terms of time efficiency, both for short and relatively straightforward data series (e.g., Bunn et al., 2005) and for longer series with and less visually clear data patterns (e.g., Abney et al., 2014).

One potential issue with the analytical method is that it is possible that, in some instances, the three components do not coincide. A cautious approach would be to gather follow-up data after a certain period of time in order to check whether the initial ambiguous result of the assessment still holds. In case the unclear change is maintained and perceived as a change by the participants, then there would be evidence in favor of its practical importance. If there is disagreement between the substantive criterion and the other two components, we think that if the clients’ well-being, quality of life, functionality, performance, etc. is improved according to their own opinion, then the substantive criterion should prevail, regardless of its numerical expression. In any case, the general effectiveness of an intervention depends on replications (Pashler and Wagenmakers, 2012) and not on the numerical result in a single study. Finally, if there is a divergence between the visual and quantitative information, it is important to know: (a) whether there is any data feature (e.g., pre-intervention trend, outliers) that might affect the performance of the quantitative analysis – in such case visual inspection should prevail; or (b) whether the data pattern prevents from getting a clear visual impression (e.g., due to highly variable data and/or a complex design structure) – in such case the quantitative summary is potentially more useful.

Another issue with the analytical method is that it might fail in certain situations such as the ones described in this paragraph (the list is not necessarily comprehensive). First, it is possible that the pre-intervention phase is too short or the measurements too variable for estimating trend with precision: the SLC quantifications would be less useful, but if there is no clear evidence of trend, then the NAP can be used as main quantification. Second, if there is complete non-overlap between the observations of the two conditions, the NAP will not be very informative, but the SLC can be used as an unstandardized quantification of the amount of difference and the *d*-statistic as a standardized quantification if more than one participant is being studied. Third, there might be a non-linear trend present in data, which is not an optimal situation for applying the SLC. In such case running medians (Tukey, 1977) can be used as a visual aid via the SCDA plug-in for R, while data modeling via the generalized least squares approach and LOESS is also possible. Fourth, there might be a delayed change in the behavior, not occurring simultaneously with the change in conditions (an issue that has remained practically unstudied except for Lieberman et al., 2010). In such case, the descriptive statistics will reflect the delay with lower quantifications of the effect, but it would be crucial to explore the cause of the change among the external uncontrolled factors (i.e., the solution is not an analytical one), given that the immediacy of the effect is one of the cornerstones for demonstrating causality (Kratochwill et al., 2010).

We hope that the discussion presented here would help practitioners and applied researchers to apply a systematic approach to data analysis and take a step toward partially improving the methodological quality of the studies. However, this would only be *one* step and studies would also need to meet the recommendations about the assessment and measurement of the target behavior, the implementation of the intervention, and the use of blinding to ensure objectivity, and also about reporting the results of the study (Tate et al., in press). Finally, it should always be considered whether what is assessed can be considered an “intervention effect” (in causal terms) or only a “behavioral change,” which after several replications might point at the possible effectiveness of the intervention. In that sense, the analytical method was described in the context of studies with less-than-optimal designs in which causal relations cannot be readily established. Nonetheless, it is possible to extrapolate the method to experimental situations (e.g., multiple-baseline designs in which it is crucial to assess whether the behavioral change coincides with the staggered introduction of the intervention).

As a limitation of the quasi-statistical component of the analytical method, it is debatable whether the numerical results can be presented confidently in absence of a conventionally accepted optimal procedure, i.e., when all analytical techniques can be criticized. Considering the analytical method as a whole, further discussion is necessary on how to proceed when practitioners are faced with data that cannot be easily analyzed visually or quantitatively (e.g., short series, great data variability). One option would be to use the substantive criteria as basis for the conclusions and label the study as “practice” but not as “research.” In contrast, when all three pieces of information (visual, quantitative, and substantive) coincide, it still has to be kept in mind that not meeting current *Standards* (Kratochwill et al., 2010) could render two-phase studies only a “pilot” status and, when included in meta-analysis, they are likely to be assigned lower weights and have less influence on the summary measures obtained.

## Author Contributions

The initial idea was due to JL and it was subsequently complemented and further developed by RM. The manuscript was written by JL (observational, non-experimental conceptual part in the Introduction) and RM (analytical part in the Analytical Method Explained, Analytical Method Applied, and Discussion). SC-M and SS-C made substantial contribution to the design of the work. All four authors (RM, JL, SC-M, and SS-C) participated in several revisions during the process of creating, discussing, and improving the manuscript, with RM leading all revisions and guiding the continuous improvement of the manuscript; gave their consent that this final version is submitted for publication; and agreed in their co-responsibility regarding all aspects of the work, such as the accuracy of the data and the integrity of the research.

## Conflict of Interest Statement

The authors declare that the research was conducted in the absence of any commercial or financial relationships that could be construed as a potential conflict of interest.

## Footnotes

1 Actually, even pre–post designs with a single measurement before and after an intervention can provide useful evidence (e.g., Pazzagli et al., 2014), especially if clinical significance is assessed, for instance using the Reliable Change Index (Jacobson and Truax, 1991).

2 http://cran.r-project.org/web/packages/RcmdrPlugin.SCDA/index.html

3 https://dl.dropboxusercontent.com/s/elhy454ldf8pij6/SD_band.R

4 https://dl.dropboxusercontent.com/s/5z9p5362bwlbj7d/ProjectTrend.R

5 Another option is to take into account the baseline data variability, operationally defined as the interquarile range, when constructing the trend stability envelope (Manolov et al., 2014).

6 Kratochwill et al. (2013) recommend that the findings be replicated in at least five different studies, conducted by at least three different research teams on a total of 20 participants or more (i.e., the 5-3-20 rule).

7 Wolery et al. (2010) found that no overlap technique had highest agreement with visual analysts for both data with and without a change. However, they did not include NAP or Tau-U (Brossart et al., 2014) in their study, and these two non-overlap indices are considered to be superior, given their more solid statistical basis and greater statistical power according to the review performed by Parker et al. (2011a).

8 This indicator is equivalent to Glass’ Δ (Glass et al., 1981), as it divides the mean difference by the standard deviation of the pre-intervention phase data.

9 We would like to thank Dr. Daniel Bagner for kindly offering the raw data for re-constructing their original figure.
